# Real-time functional magnetic resonance imaging neurofeedback in motor neurorehabilitation

**DOI:** 10.1097/WCO.0000000000000340

**Published:** 2016-05-19

**Authors:** David E.J. Linden, Duncan L. Turner

**Affiliations:** a MRC Centre for Neuropsychiatric Genetics and Genomics, Cardiff University School of Medicine, and Cardiff University Brain Imaging Centre, Cardiff; b Neurorehabilitation Unit, School of Health, Sport and Bioscience, University of East London, London, UK

**Keywords:** neurological recovery, neuroplasticity, Parkinson's disease, real-time functional magnetic resonance imaging, stroke

## Abstract

**Purpose of review:**

Recent developments in functional magnetic resonance imaging (fMRI) have catalyzed a new field of translational neuroscience. Using fMRI to monitor the aspects of task-related changes in neural activation or brain connectivity, investigators can offer feedback of simple or complex neural signals/patterns back to the participant on a quasireal-time basis [real-time-fMRI-based neurofeedback (rt-fMRI-NF)]. Here, we introduce some background methodology of the new developments in this field and give a perspective on how they may be used in neurorehabilitation in the future.

**Recent findings:**

The development of rt-fMRI-NF has been used to promote self-regulation of activity in several brain regions and networks. In addition, and unlike other noninvasive techniques, rt-fMRI-NF can access specific subcortical regions and in principle any region that can be monitored using fMRI including the cerebellum, brainstem and spinal cord. In Parkinson's disease and stroke, rt-fMRI-NF has been demonstrated to alter neural activity after the self-regulation training was completed and to modify specific behaviours.

**Summary:**

Future exploitation of rt-fMRI-NF could be used to induce neuroplasticity in brain networks that are involved in certain neurological conditions. However, currently, the use of rt-fMRI-NF in randomized, controlled clinical trials is in its infancy.

## INTRODUCTION

Over the last quarter of a century, functional magnetic resonance imaging (fMRI) has become an important tool for the noninvasive monitoring of neural activity in human participants undertaking a wide range of behaviours and altered neural activity in neurological diseases such as Parkinson's disease (PD) and stroke. fMRI measures changes in the blood oxygen level dependent (BOLD) signal and thus provides a surrogate measure of neural activity. With the advent of increasingly fast processing tools, it has become possible to measure changes in task-related BOLD signal, for example during a hand grasp, and thus neural activity of a ‘motor task network’, on a quasireal-time subsecond basis (more advanced processing of raw data for large network connectivity measures still takes 1–5 s in practice) [1]. The real-time representation of brain network activity or connectivity can be relayed to an observer via visual display. The observer is almost always the investigator and enables better control over experiments, body motion during scanning and updating of fMRI data, for example [1,2].

A key conceptual switch was to offer the visual representation of the neural activity back to the participant thereby affording him/her feedback of his/her own brain activity. Now the participant is, in principle, able to learn how to regulate complex neural activity in his/her own central nervous system; in practice, this is often achieved by the participant varying a simplified representation of the complex brain activity pattern such as a virtual thermometer. The crucial instructional behavioural component of the real-time fMRI-based neurofeedback (rt-fMRI-NF) technique is that the participant is asked to undertake a task such as motor imagery of hand movements in order to vary the thermometer height. The task he/she undertakes to vary the thermometer height is explicitly contingent on varying the BOLD signal in a brain target region of interest, localized beforehand by asking the participant to perform real hand grasps in our example here. This localizer target region, that is a region in the motor network for hand grasps, is often part of the neural network activated in the self-regulation task, in this case motor imagery. Thus, the participant is now able to learn how to self-regulate the BOLD signal and presumably neural activity in a specific region or network.

Since the first demonstrations of rt-fMRI-NF at the beginning of the century, there has been an expanding literature describing increasingly elaborate use of rt-fMRI-NF, although the fundamental methodology has remained relatively unaltered [3^▪^,^4^–^8^]. The range of uses of rt-fMRI-NF includes self-regulation of brain regions or networks involved in a number of behavioural repertoires such as emotional control [9–14], pain control [15], auditory and visual performance [16–19], attention [20], memory performance [21–24], reward [25,26] and motor control [27–35] in healthy individuals. Of particular note, however, is that rt-fMRI-NF appears to be differentially effective across different target regions of the brain, for example the extrinsic (visual) and intrinsic (default mode network) nodes of the cortex [36] – we will return to this important point.

One important distinction in the implementation of neurofeedback protocols is whether purely to rely on instrumental learning, as in most electroencephalography-neurofeedback studies, or whether to use specific instructions or suggest strategies that participants might employ to achieve self-regulation. An example of the latter approach would be providing information about the functional role of the target area and suggesting kinaesthetic imagery as a potential strategy in the example of motor cortex upregulation introduced above. This approach has the advantage of potentially accelerating training, which can be helpful for adherence in patient studies and reducing costs, particularly when using expensive imaging equipment. Its disadvantages are that it may overly constrain participants’ strategies – and then actually counteract training success – and restrict target areas to those with a well-documented and circumscribed functional role.

The next deployment of rt-fMRI-NF could clearly be directed toward neurological conditions and to developing novel neurorehabilitation strategies, whereby the patient learns to self-regulate brain activity in an injured/disordered region or network. Here, we focus on control of motor execution and motor skill learning to illustrate a translational framework for exploiting rt-fMRI-NF in motor neurorehabilitation.

A key initial step is to demonstrate that rt-fMRI-NF can lead to regulation of brain regions which are thought to be involved in motor execution and skill learning. The next step is to demonstrate that learned self-regulation of neural activity leads to a sustained change in brain activity, for example that patients with a brain injury can reactivate injured regions or upregulate other regions to compensate for neuronal damage. A further important step is to demonstrate that rt-fMRI-NF leads to a clinically important change in impaired behaviour such as clinical motor impairment of hand grasping and gait in our illustrated neurological conditions. There are many design-based, mechanism-based and clinical-based factors that are yet to be addressed in translating the fundamental model of rt-fMRI-NF to appropriately designed clinical trials, and we will highlight some important caveats. 

**Box 1:**
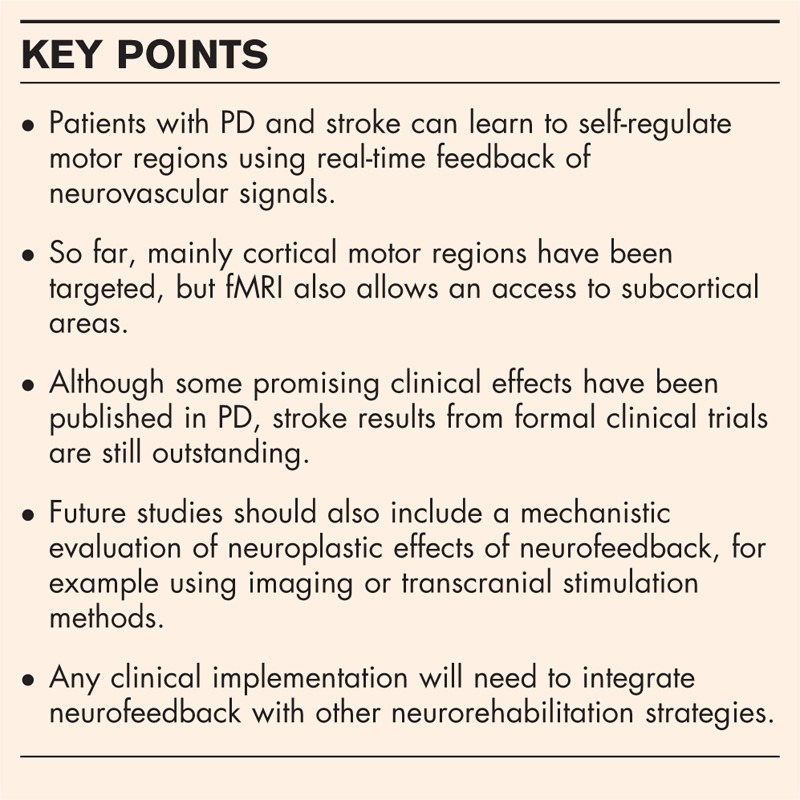
no caption available

## REAL-TIME FUNCTIONAL MAGNETIC RESONANCE IMAGING NEUROFEEDBACK IN HEALTHY INDIVIDUALS: MODULATING BLOOD OXYGEN LEVEL DEPENDENT SIGNAL IN MOTOR-RELATED REGIONS OR NETWORKS

Execution of complex movements such as reaching, grasping or locomotion involves a distributed network of brain regions including primary motor cortex (M1), supplementary motor area (SMA), premotor cortex (PMC), parietal cortex, basal ganglia, cerebellum, brainstem and spinal cord [37–39]. Motor performance is optimized by integrating and transforming sensory feedback signals, mainly visual and kinesthetic inputs related to movement preparation and execution, through visual and extrastriate pathways, spinal cord, thalamus and somatosensory cortex (SSC) [40–42]. Within these networks, meta-analysis using activation likelihood estimates (ALEs) of learning novel motor skills has highlighted significant roles for dorsal PMC, SMA, M1, SSC, superior parietal lobule (SPL), thalamus, and putamen of the basal ganglia and cerebellum [43]. Thus, on the face of it, applying rt-fMRI-NF to regulate neural activity in one or combinations of these regions or the connectivity between regions could enhance motor control, performance and skill learning. This would be an important first step to facilitate the processes of neuroplasticity, which are thought to operate in highly skilled motor performers and during recovery from brain injury [44–47].

One approach to choosing the ‘correct’ target region(s) for rt-fMRI-NF might be to contrast the sensorimotor network required for execution and skill learning against a similar network for motor imagery, because this is the preferred method used to date to learn self-regulation of motor execution-related regions [27–35]. A meta-analysis using ALE suggested that motor imagery activates several large clusters spanning over both hemispheres, with considerable overlap with the motor control network described above [48]. Consistently activated regions during motor imagery include bilateral inferior frontal gyri, precentral gyrus anterior to motor cortex (i.e. PMC), middle frontal gyrus, SMA and regions of the anterior insula. In the parietal lobes, the bilateral SPL and supramarginal gyrus were also consistently activated. Subcortical regions included the left putamen (basal ganglia), right thalamus and area VI (bilateral) and the vermis of the cerebellum.

However, contrasting ALE-based meta-analyses for motor execution and learning with motor imagery highlights a notable absence of consistent M1 activity during motor imagery compared with execution. This may explain why it has proven difficult to use the M1 as a single target region for learned self-regulation in some rt-fMRI-NF paradigms to date [27–35,49]. Improved learned self-regulation has been demonstrated, however, when both ipsilateral and contralateral motor cortices are combined targets [50]. Self-regulation of the BOLD signal can also be achieved in the ventral PMC with rt-fMRI-NF [34] and, interestingly, the individual rate of learned self-regulation in PMC was linearly related to baseline intracortical facilitation within circuits in M1 (i.e. in the motor execution network) measured with transcranial magnetic stimulation [51]. This may be a result of the extensive connections between PMC and M1 as self-regulation of PMC as a single target can also lead to increased connectivity in the wider motor imagery network and in particular is related to the initial strength of PMC-parietal cortex connectivity before the rt-fMRI-NF training programme [31]. The current evidence suggests that using a known single target region involved in motor imagery for rt-fMRI-NF can lead to changes in cortical network connectivity, which may also include M1, even though M1 is not activated robustly by motor imagery per se. In other words, there may be indirect paths to target M1 function and thus motor execution via, for example rt-fMRI-NF of PMC [52,53,54^▪^]. This may have profound impact for the development of rt-fMRI-NF protocols for neurological conditions wherein motor cortex or inputs to motor cortex are damaged.

## REAL-TIME FUNCTIONAL MAGNETIC RESONANCE IMAGING NEUROFEEDBACK IN HEALTHY INDIVIDUALS: SUSTAINING BRAIN ACTIVITY AFTER SELF-REGULATION TRAINING HAS STOPPED

In some rt-fMRI-NF studies, a single follow-up ‘transfer’ session has been conducted at a time point after the neurofeedback sessions to test for sustainable modulation of brain activity. The transfer session consists of testing the ability to self-regulate a target region but without the feedback component. Four weeks (12 sessions) of rt-fMRI-NF resulted in a significant transfer effect after targeting the contralateral and ipsilateral M1 BOLD signal difference [50]. The measure taken to represent sustainable impact of rt-fMRI-NF can also be modulation of brain networks using resting-state fMRI, wherein sustainable changes were demonstrated one day after the end of self-regulation training [55]. Finally, when the rt-fMRI-NF sessions are followed up with similar behavioural practice (with no explicit feedback) for 2 weeks, then sustained changes in motor execution networks can occur compared with no behavioural practice suggesting a possible role of rt-fMRI-NF in facilitating motor skill learning – although the performance was only rated subjectively and not by objective means in this case [49]. Although these initial findings are promising, sustainability of the effects of rt-fMRI-NF requires more substantiation in future.

## REAL-TIME FUNCTIONAL MAGNETIC RESONANCE IMAGING NEUROFEEDBACK IN HEALTHY INDIVIDUALS: MODULATING BEHAVIOUR IN AN APPROPRIATE MANNER

The current evidence would suggest that rt-fMRI-NF based on motor imagery is most effective when it is used to modulate activity (BOLD signal) in target regions outside M1. The next challenge is to show that this neurofeedback training (or neurofeedback-enhanced imagery training) can result in appropriate changes in behaviour such as motor performance and skill learning in healthy individuals. Upregulation of M1 with rt-fMRI-NF did not change simple finger reaction time [29]. However, in another study, the degree of upregulation of BOLD in M1 during motor imagery correlated with motor performance (pinch force) [28] suggesting that subject-by-subject variability in motor imagery abilities (and ability to change BOLD per se) may have an important role in successful changes in motor performance.

Upregulation of left ventral PMC BOLD signal may reduce ipsilateral, intracortical inhibitory drive in M1, but this neural response to rt-fMRI-NF did not transfer to a better response in a visuomotor tracking task [51]. Upregulation of right PMC BOLD signal was correlated with the degree of functional interhemispheric PMC-parietal cortex connectivity during motor imagery and right cerebellum to left M1 connectivity during right hand motor execution. These changes were associated with an increase in maximal finger tapping speed using the right hand [31]. When the SMA is used as a target region for rt-fMRI-NF with motor imagery, the individual ability to upregulate the BOLD signal in SMA was correlated with only a small reduction in motor reaction time [35]. Therefore, there appear to be rather more complex relationships between rt-fMRI-NF and motor performance than merely a simple cause–effect between regulation of the neural target and behaviour. The neural correlates of rt-fMRI-NF-induced changes in appropriate behavioural motor performance require more investigation. Moreover, the role of rt-fMRI-NF in enhancing motor skill learning has not been studied to date, beyond subjective self-reporting of improved performance [49]. This would seem to be an essential challenge to overcome if rt-fMRI-NF is to be used to activate neuroplastic mechanisms of recovery in neurological conditions affecting the motor system.

## REAL-TIME FUNCTIONAL MAGNETIC RESONANCE IMAGING NEUROFEEDBACK IN PATIENTS WITH MOTOR DISORDERS: PARKINSON'S DISEASE AND STROKE

The evidence for clinical effects of rt-fMRI-NF in neurological motor conditions is currently limited to data from small proof-of-principle studies and small randomized trials [56].

Two small studies have explored the feasibility of rt-fMRI-NF in PD. Five patients in the early stages of PD (Hoehn and Yahr stages I–III) were trained in the upregulation of activity in the SMA over two sessions, separated by 2–6 months [57]. As compared with a control group engaged in motor imagery without feedback, the patients in the rt-fMRI-NF group achieved higher activation levels, showed improved motor fluidity (as assessed by finger-tapping speed) and improved on the motor scale of the Unified PD Rating Scale, a standard scale for the assessment of changes in motor symptoms over time. Because of the long interval between the rt-fMRI-NF sessions, patients were instructed to practice their imagery strategies regularly at home. This requirement for the home-based practice highlights one of the challenges – as well as opportunities – of rt-fMRI-NF; because of the cost and availability of the equipment, only a relatively small number of sessions will be available, which entails the need for appropriate ‘homework’ and therefore real-life transfer protocols. A study using a similar protocol in a single patient (and three healthy volunteers) confirmed feasibility of upregulation of SMA although responses on a sequenced motor task were slower after the training [58]. Larger studies with combinations of quantitative motor assessments and standardized clinical scales are clearly needed.

One open question is whether upregulation of the SMA is the most appropriate protocol for PD. Although some studies have reported hypoactivation of SMA in PD, others have reported hyperactivation, and regional differences in activation have been observed within the SMA itself [56]. Further work, looking at the stage-dependence (and state-dependence) of SMA activation patterns and intraarea differences, may therefore be needed in order to inform the selection of targets for self-regulation training in PD. It may be the case that the optimal protocol selection will vary across stages of the disease. Beyond the choice of the ‘correct’ target area (or subregion), other parameters that can be varied include the level of desired upregulation and the multivariate activation pattern. Furthermore, the choice of target area can be influenced by the mechanistic model of the intervention. In the case of PD, one might aim to influence the regions most directly affected by the pathophysiological process, such as the substantia nigra [26], or circuits that potentially support compensatory mechanisms [59].

In stroke, unlike PD, the lesion(s) that leads to motor impairment may occur in a variety of cortical and/or subcortical regions and brainstem and may vary in size. Thus, the choice of neural targets for rt-fMRI-NF in order to improve motor impairment becomes more difficult. Nevertheless, there are proof-of-principle studies using rt-fMRI-NF in stroke recovery.

Two chronic subcortical stroke patients with only mild upper limb motor impairment (Fugl–Meyer scores of 55/66 and 60/66, where a score of 66 is full use of the upper limb) were trained over three sessions per day for 3 days with rt-fMRI-NF using self-chosen strategies to upregulate BOLD signal in the ventral PMC. The stroke patients were able to upregulate ventral PMC to a similar degree as healthy individuals and upregulation persisted during an fMRI task of motor imagery without feedback immediately after training although visuomotor tracking behaviour was not consistently improved [51].

In another small feasibility study, four moderate-to-severe upper limb motor-impaired patients with cortical or cortical and subcortical first-ever stroke lesions (Fugl–Meyer scores of 13–28/66) undertook motor imagery-based rt-fMRI-NF focussing on imagining movement in the affected hand [60]. The target in this case was ipsilesional primary motor cortico-thalamic connectivity measures rather than the single neural target approach of, for example, the PMC. Three from four patients were able to upregulate ipsilesional cortical-subcortical connectivity successfully and maintain the effect, while imagining without feedback immediately after the last rt-fMRI-NF training session. There were no behavioural performance measures undertaken, and there were no clinical outcome measures employed post rt-fMRI-NF training to test for clinically significant improvements.

The largest study using real-time neurofeedback in stroke recovery used an alternative but conceptually similar method to fMRI, namely, near-infra red spectroscopy (fNIRS) for relaying feedback of task-related regional hemodynamic changes in oxygenated hemoglobin [61,62^▪^]. Patients were at least 12 weeks post their first-ever stroke with moderate-to-severe motor impairment (an average Fugl–Meyer score of 21). The PMC was used as a single neural target region for rt-fNIRS-NF over six sessions of 20 min of imagery of distal hand/finger motor tasks over 2 weeks. The 10 stroke patients who received ‘real’ neurofeedback of ipsilesional PMC activation during imagery could significantly upregulate the oxygenated hemoglobin signal, whereas the 10 patients receiving ‘sham’ neurofeedback (irrelevant randomized visual signals) did not. Importantly, the level of rt-fNIRS-NF induced activation in PMC was correlated with an improvement in the finger (distal) subscale of the Fugl–Meyer score, but not the more proximal subscale suggesting an imagery task-related relationship between rt-fNIRS-NF and clinical improvements. Taken together, the preliminary evidence suggests that rt-fMRI-NF may be useful in improving clinical measures of motor impairment in stroke recovery. The neural mechanisms of action and long-term retention of self-regulated regions or networks involved in stroke recovery remain to be explored.

## REMAINING CHALLENGES AND FUTURE QUESTIONS

Choosing the ‘correct’ target region or connection for rt-fMRI-NF will likely differ between different neurological motor conditions. A framework for considering possible targets might be enhanced by considering each condition as a ‘network’ problem. Recent studies have analyzed network connectivity on a brain-wide level in PD patients and correlated motor symptoms to path-specific hypoactivity and hyper involving basal ganglia, SMA and wider fronto-parietal networks [63–65]. Similarly, in stroke, there is now ample evidence to demonstrate that a focal lesion can lead to disruption of connectivity across the motor execution network and indeed beyond [45,66–68]. This has led to rethinking how to improve motor function by (re)activating higher motor targets that can then either impact on the damaged connections and/or enhance healthy regions in order to compensate behaviour in the light of established damage [52,53,54^▪^,^57^,62^▪^].

Thus far, we have assumed that the ‘core’ network for self-regulation of brain activity is operative both in PD and stroke. A recent meta-analysis of rt-fMRI-NF to date suggested that this core network involved in rt-fMRI-NF independent of target region included mainly the anterior insula and basal ganglia [69]. If either or both of these regions are impacted by PD or stroke, then success in using rt-fMRI-NF might be compromised.

Likewise, motor imagery is a common behavioural tool with which to self-regulate brain regions especially in the motor network. It is known that motor imagery can be disrupted or become chaotic after a stroke and PD [70–72] and that poor motor imagery vividness is associated with lesions in the left putamen (i.e. basal ganglia), left ventral PMC and long association fibers linking parieto-occipital regions with the dorsolateral premotor and prefrontal areas [73]. Furthermore, while motor execution network connectivity can recover in well-recovered stroke patients, the motor imagery network connectivity may not always recover in synchrony [74]. Thus, motor imagery brain networks may be disrupted in motor conditions and this might impact on the effectiveness of using motor imagery as a tool in rt-fMRI-NF. On the contrary, there is some promising evidence suggesting that rt-fMRI-NF can lead to self-regulation of brain activity in both PD [57] and stroke [60,62^▪^].

## CONCLUSION

Neurofeedback using fMRI or other neurovascular techniques has good conceptual validity for neurorehabilitation, because it can support the internal activation of compensatory processes and may aid the restitution of damaged brain tissue but its evaluation is still in its infancy. Both mechanistic and clinical studies are needed to evaluate the potential of neurofeedback to promote neuroplasticity and aid functional recovery. Studies should also evaluate whether neurofeedback in one domain, for example targeting motor networks, may have associated benefits in other domains, for example on cognitive functions or vice versa [75].

Ultimately, any clinical application will have to integrate neurofeedback into a wider framework of neurorehabilitation interventions. If evidence for clinical efficacy can be provided, then the next challenge will be to justify the costs of neurofeedback, especially for protocols based on the relatively costly fMRI technique, but in view of the considerable socioeconomic and healthcare costs of PD [76], stroke [77] and other motor disorders, this may be feasible.

## Acknowledgements

*None*.

### Financial support and sponsorship

This work was supported by an MRC Confidence in Concept grant.

### Conflicts of interest

*The authors conduct research on functional imaging-based neurofeedback interventions for neurological diseases, which are currently purely investigational, but have no financial conflict of interest in relation to this work*.

## REFERENCES AND RECOMMENDED READING

Papers of particular interest, published within the annual period of review, have been highlighted as:

▪ of special interest
▪▪ of outstanding interest
